# Supplementary material for: Do You Know What You Eat? Kebab Adulteration in Poland
Source: Foods. 2023 Sep 9;12(18):3380. doi: 10.3390/foods12183380 (PMC10530059; doi:10.3390/foods12183380)
Supplement: Supplementary file 1 [file foods-12-03380-s001.zip › foods-2577887-supplementary.pdf]

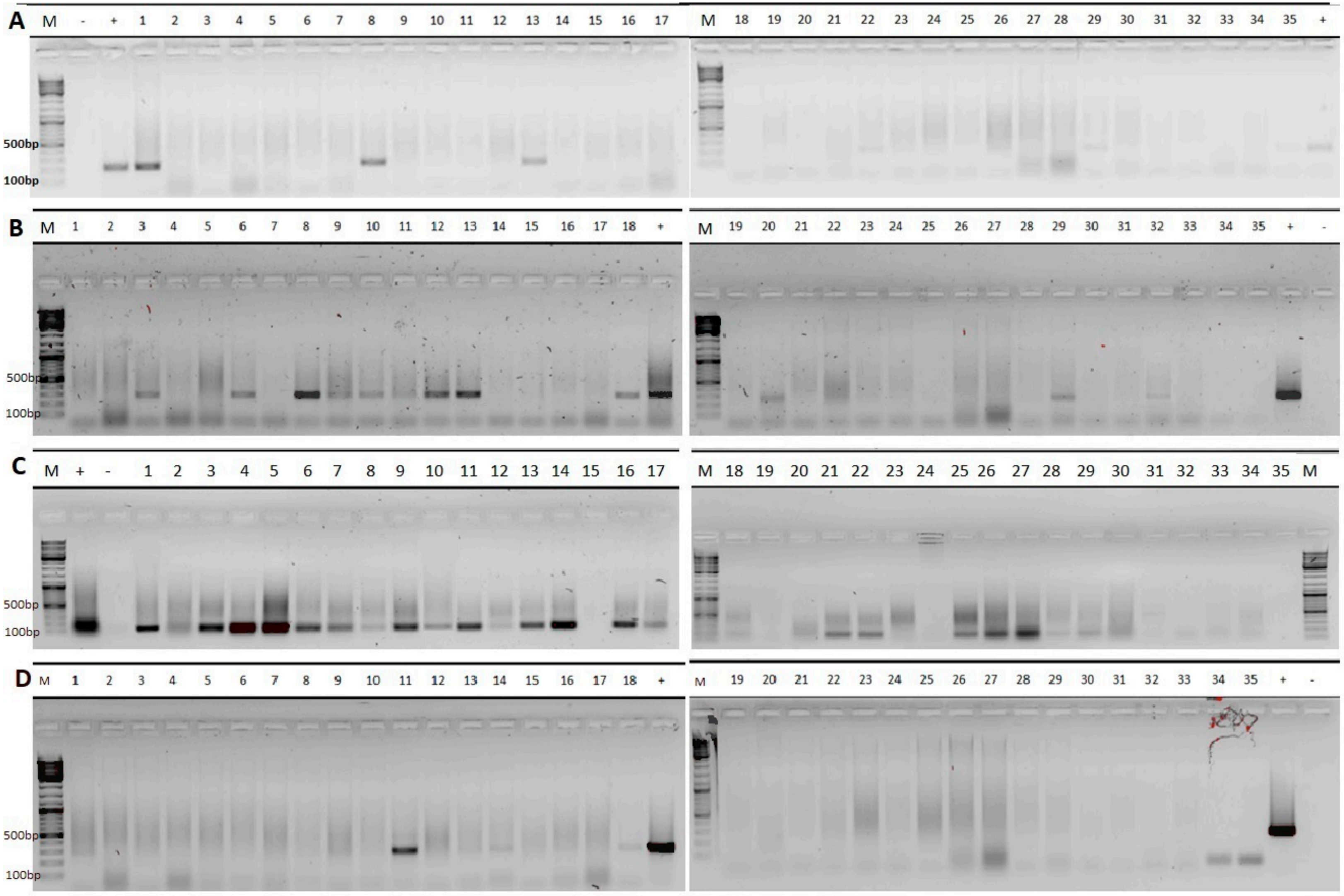

Figure S1. Electrophoretic analysis of amplicons from DNA obtained from Kebab samples (1-35) using species-specific primers for lamb (**A**), cow (**B**), chicken (**C**), and pig (**D**). M molecular weight marker (Thermo Scientific Gene Ruler DNA Ladder Mix),"+"- positive control, "-" - no-template control.
